# Influence of 2′-Fucosyllactose and *Bifidobacterium longum* Subspecies *infantis* Supplementation on Cognitive and Structural Brain Development in Young Pigs

**DOI:** 10.3389/fnins.2022.860368

**Published:** 2022-04-25

**Authors:** Loretta T. Sutkus, Sangyun Joung, Johanna Hirvonen, Henrik Max Jensen, Arthur C. Ouwehand, Ratna Mukherjea, Sharon M. Donovan, Ryan N. Dilger

**Affiliations:** ^1^Neuroscience Program, University of Illinois at Urbana-Champaign, Champaign, IL, United States; ^2^IFF Health and Biosciences, Kantvik, Finland; ^3^IFF R&D—Enabling Technologies, Advanced Analytical, Brabrand, Denmark; ^4^IFF Health and Biosciences, St. Louis, MO, United States; ^5^Department of Food Science and Human Nutrition, University of Illinois at Urbana-Champaign, Champaign, IL, United States; ^6^Department of Animal Sciences, University of Illinois at Urbana-Champaign, Champaign, IL, United States

**Keywords:** brain, neurodevelopment, behavior, magnetic resonance imaging, 2′-fucosyllactose, *Bifidobacterium infantis* Bi-26

## Abstract

Development of the gut-brain axis during early-life is an important contributor of brain structural and functional development. Human milk oligosaccharides and gut microbiota have potential beneficial effects on various aspects of development; however, the effects of 2′-fucosyllactose (2′-FL) and *Bifidobacterium longum* subsp. *infantis* Bi-26 (Bi-26) administration during infancy separately and combined are still not clear. Therefore, we investigated the effects of early administration of dietary 2′-FL and Bi-26 on brain structural and functional development in the young pig. From postnatal day (PND) 2–34 or 35, fifty-two intact male pigs were randomly assigned to treatment groups in a 2 × 2 factorial arrangement and provided *ad libitum* access to a nutritionally adequate milk replacer without or with 1.0 g of 2′-FL/L of reconstituted liquid. Pigs within each diet group were further stratified to receive a daily oral dose of glycerol stock without or with Bi-26 (10^9^ CFU). Pigs were subjected to the novel object recognition (NOR) task from PND 27–31 to assess recognition memory and subsequently underwent magnetic resonance imaging procedures at PND 32 or 33 to assess brain macrostructure and microstructure. Pigs that received Bi-26 had smaller absolute brain volumes for 9 of 27 brain regions of interest, and smaller relative volumes for 2 regions associated with kinesthesia (*P* < 0.05). Synbiotic administration of 2′-FL and Bi-26 elicited interactive effects (*P* < 0.05) on several microstructural brain components, where dual supplementation negated the effects of each test article alone. Behavioral outcomes indicated that pigs did not express novelty preference, regardless of treatment group, demonstrating no effects of 2′-FL and Bi-26 on recognition memory when supplemented alone or in combination. Interactive effects (*P* < 0.05) were observed for the number of all object visits, latency to the first object visit, and number of familiar object visits. Pigs that did not receive Bi-26 supplementation exhibited less time interacting with the familiar object in total (*P* = 0.002) and on average (*P* = 0.005). In conclusion, supplementation of 2′-FL and/or Bi-26 elicited some alterations in object exploratory behaviors and macro/micro-structures of the brain, but changes in recognition memory were not observed. Specifically in brain microstructure, synbiotic administration of 2′-FL and Bi-26 appeared to negate effects observed when each dietary article was supplemented separately.

## Introduction

The gut microbiome is essential for establishing a healthy gut and ensuring absorption of nutrients to provide energy for proper development (Moore and Townsend, 2019). The gut-brain axis is the bi-directional communication between the gut and the brain with several mediators including the gut microbiota and their metabolites, the immune system, the autonomic nervous system, and several brain circuitries (Sudo et al., 2004; Barbara et al., 2005; Carabotti et al., 2015; Sherman et al., 2015; Kim et al., 2018). A healthy gut-brain axis development is associated with proper brain and behavioral development in animal species (Carabotti et al., 2015; Foster, 2016; Jena et al., 2020), as evidenced by behavioral, neuro-morphological and neurochemical deficits observed in germ-free mice (Sudo et al., 2004; Neufeld et al., 2011; Clarke et al., 2013; Luczynski et al., 2016). Therefore, the importance of normative development of the gut-brain axis and the gut microbiota during early-life are crucial for normal brain structural and behavioral development.

Diet is an important factor influencing gut microbial diversity and their metabolites. Human milk is the optimal nutritional source for infants to support health and development (Bode, 2012). Human milk contains high concentrations of human milk oligosaccharides (HMOs), which are resistant to gastrointestinal digestion; thus, they reach the large intestine, where they are metabolized by gut microbiota, especially *Bifidobacterium* strains, as energy sources (Asakuma et al., 2011; Underwood et al., 2015). In particular, *Bifidobacterium longum* subsp. *infantis* (*B. infantis*), is abundant in the feces of breastfed infants due to their ability to help metabolize HMOs, including 2′-FL (Underwood et al., 2015; Rivière et al., 2016). This is true for other species as well, where the presence of 2′-FL was observed in porcine milk at different stages of lactation, indicating that pigs naturally consume 2′-FL and the corresponding *B. infantis* species were also present in the swine gut microbiota (Heinritz et al., 2013; Mudd et al., 2016). Previous research has elucidated how *B. infantis* can metabolize fucosylated HMOs such as 2′-FL (Yu et al., 2013; Thomson et al., 2018).

Consumption of HMOs during early-life, specifically 2′-FL, alters various modulators of the gut-brain axis, including lowering pro-inflammatory immune responses, increasing bifidobacteria abundance in the gut, and improving the gut barrier integrity and vagal afferent signaling (Goehring et al., 2016; Cerdo et al., 2017; Berger B. et al., 2020; Lee et al., 2021). Additionally, numerous animal and clinical studies have demonstrated the long-term beneficial effects of 2′-FL consumption during early-life in terms of cognitive development (Oliveros et al., 2016; Berger P. K. et al., 2020) and potential mechanisms for brain structural changes (Vázquez et al., 2015). The relationship between 2′-FL and *B. infantis* plays a crucial role in the gut-brain axis, and *Bifidobacterium* species supplementation enhances performance on various cognitive tasks assessing different aspects of learning and memory in several animal and human clinical studies (Savignac et al., 2015; Allen et al., 2016; Talani et al., 2020). Together, these findings suggest beneficial effects of early-life 2′-FL and *B. infantis* supplementation on brain and cognitive development, and that the pig may serve as a valuable translational model to investigate their effects.

Due to the plethora of beneficial outcomes associated with prebiotics and probiotics separately, studying their synbiotic administration is of increasing interest (Collins and Gibson, 1999). Previous research has shown that synbiotics are associated with beneficial cognitive outcomes in rats with diabetic conditions, though research has been scarce in this field (Chunchai et al., 2018; Morshedi et al., 2020). Additionally, when paired with glutamine, synbiotics improved brain maturation in preterm pigs (Andersen et al., 2019). These results indicate that structural and behavioral outcomes may arise from synbiotic treatment, but there are still many unknowns regarding the full developmental impact of synbiotics, especially during infancy. In the present study, the combination of 2′-FL and *B. infantis* Bi-26 (Bi-26) was chosen, because of their established relationship in the gut and their impact separately on cognitive development.

The pig was chosen as a preclinical model due to similarities in nutritional requirements, structural intestinal development and neurological development (Sauleau et al., 2009; Conrad et al., 2012; Heinritz et al., 2013; Hoffe and Holahan, 2019; Lunney et al., 2021). Given the natural presence of 2′-FL and Bi-26 in the pig, as well as the numerous advantages as a preclinical model, the objective of the current study was to investigate the effects of individual and synbiotic supplementation of 2′-FL and Bi-26 on brain structural and functional development during early-life using young pigs.

## Materials and Methods

### Animal Care and Housing

All animal care and experimental procedures listed were approved by the University of Illinois Urbana-Champaign Institutional Animal Care and Use Committee and in accordance with the National Research Council Guide for the Care and Use of Laboratory Animals. A total of fifty-two intact (i.e., not castrated) male pigs were obtained from a genetics multiplier herd at postnatal day (PND) 2 and artificially reared at the University of Illinois Piglet Nutrition and Cognition Laboratory (PNCL) until PND 34 or 35. To prevent variability across individuals due to genetic makeup, pigs were obtained from PIC Line 3 sires. PIC Line 3 dams were artificially inseminated using a pooled semen source from 50–150 boars to ensure that pigs within and between litters were genetically similar. The study was completed using 5 separate cohorts consisting of 12 pigs each. Pigs were randomly assigned to four different treatment conditions, described below (section “Experimental Treatment Groups”), based on their initial body weights and litter of origin. Four pigs were excluded from behavioral testing and brain imaging and were only used to assess gastrointestinal development and function (Daniels et al., 2022).

Pigs were individually housed in custom pig rearing units (87.6 cm × 88.9 cm × 50.8 cm; L × W × H) as described previously (Fil et al., 2021a). Each caging unit was composed of vinyl-coated metal flooring, a stainless-steel wall and three acrylic walls to allow pigs to see, hear, and smell, but not touch neighboring pigs. The rearing environment was maintained on a 12 h light and dark cycle with ambient temperature set at 26.6°C during the duration of the study. Animal health observations were recorded twice a day to monitor any clinical indicators such as diarrhea, lethargy, weight loss, or vomiting.

### Experimental Treatment Groups

The control diet was a commercial milk replacer (ProNurse^®^ Specialty Milk Replacer, Land O’Lakes, North Arden Hills, MN, United States) supplemented with 0.532% lactose to match the same percentage of 2′-FL (CARE4U™, International Flavors and Fragrances, New York, NY, United States) supplementation in the test diet. Both control and test milk replacers were prepared in powder form (TestDiet, St. Louis, MO, United States) and the complete composition of the diets and test articles has been described previously (Daniels et al., 2022). Milk replacer treatments were reconstituted fresh daily at 200 g of powder per 800 g of tap water. Pigs were provided *ad libitum* access to liquid diets within a 20-h daily feeding cycle from 10:00 h to 06:00 h the following day from PND 2 to PND 34 or 35. Milk disappearance, body weight, and health observations were captured daily, and the overall health outcomes of pigs were reported separately (Daniels et al., 2022).

In addition to the milk replacer treatments, pigs were further stratified to receive a daily oral dose of glycerol stock without or with *B. infantis* Bi-26 (Bi-26, ATCC SD 6720, FloraFIT^®^ Probiotics; Danisco United States Inc., Madison, WI, United States). Bi-26 at a dose of 10^9^ CFU/pig/d was solubilized in bacterial glycerol stock (12.1% glycerol), frozen at −80°C for storage, and thawed each day before administration. Pigs were either orally administered with Bi-26 or an equal dose of glycerol stock through a syringe from PND 2–12, after which time the mixture was added to 10 mL of each pig’s assigned milk replacer treatment prior to the initial delivery of fresh milk each day (PND 13–34 or 35).

The 4 treatment groups included the following (group designations denoted): (1) control without supplementation (CON; *n* = 12), (2) 2′-FL alone (FL; *n* = 14), (3) Bi-26 alone (BI; *n* = 14), and (4) a combination of 2′-FL and Bi-26 (FLBI; *n* = 12). All personnel remained blinded to dietary treatment identity throughout the duration of the study and analyses.

### Behavioral Testing

Novel object recognition (NOR) task, previously described in detail (Joung et al., 2020; Fil et al., 2021a), was used to assess object recognition memory and exploratory behaviors as indicators of brain functioning of the pigs. NOR task consisted of four phases: a habituation, sample, delay and test phases. During the habituation phase (PND 27–28), each pig was placed in an empty testing arena for 10 min each day for two consecutive days. In the sample phase (PND 29), pigs returned to the testing arena with two identical objects placed in the center and were allowed to explore for 5 min. After a delay of 48 h, the test phase (PND 31) was conducted with one familiar object and one novel object in the arena, and the pigs were allowed in the arena for 5 min for exploration. Between trials, the objects were removed from the testing arena and immersed in hot water with detergent to mitigate odor, and the arena was sprayed with water to remove urine and feces from the previous animal. The object pair chosen for the NOR task were previously validated to have no innate preference for one over the other (Fleming and Dilger, 2017). The objects had a range of characteristics (i.e., color, texture, shape, and size), however, the novel and sample objects only differed in shape and size. The recognition index, the proportion of time spent with the novel object compared to total exploration of both objects, was compared to a chance performance value of 0.50 to assess recognition memory. A recognition index of 0.50 was considered a chance performance level due to the task having just two objects each time a pig is introduced into the arena. A recognition index greater than 0.50 was interpreted as a subject displaying novelty preference, and thus, recognition memory.

### Magnetic Resonance Imaging

Pigs underwent magnetic resonance imaging (MRI) procedures at PND 32 or 33 at the Beckman Institute Biomedical Imaging Center (Champaign, IL, United States) using a Siemens MAGNETOM Prisma 3T MRI. The pig neuroimaging protocol included a magnetization prepared rapid gradient-echo (MPRAGE) sequence to assess macrostructure and diffusion tensor imaging (DTI) technique to assess brain microstructure. Pigs were immobilized by inducing anesthesia through an intramuscular injection of a telazol:ketamine:xylazine solution [50.0 mg tiletamine plus 50.0 mg of zolazepam reconstituted with 2.50 mL ketamine (100 g/L) and 2.50 mL xylazine (100 g/L); Fort Dodge Animal Health, Overland Park, KS, United States] at 0.03 mL/kg of body weight. Anesthetized pigs were placed in a supine position in the MRI machine with the head fitted into a custom made 8-channel pig head coil. To maintain anesthesia, inhalation of isoflurane was administered at 2% and oxygen levels were monitored to be 98% or higher throughout the entire procedure. Two pulse oximeters (LifeWindow LW9x, Boynton Beach, FL and MEDRAD Veris 8600, Indianola, PA, United States), each with an infrared sensor, were clipped onto the pig’s tail and/or left hind foot pad to monitor heart rate and oxygen levels. Heart rate, partial pressure of oxygen (PO_2_), and percent isoflurane were recorded every 5 min beginning after anesthetic induction to assess overall well-being of the pig. Total scan time for each pig was approximately 45 min.

#### Structural Magnetic Resonance Imaging Acquisition and Analysis

Anatomic images of the pig brain were obtained through a 3D T1-weighted MPRAGE sequence with 0.6 mm isotropic voxel size across the entire head from the tip of the head to the cervical/thoracic spinal cord junction. The following specific parameters were used: repetition time = 2,000 ms; echo time = 6.5 ms; inversion time = 1,060 ms, flip angle = 9°, matrix = 256 × 256; slice thickness = 0.60 mm. Image processing involved manually extracting pig brains using FMRIB Software Library (FSL, RRID:SCR_002823). After extraction, individual images were manually oriented using imaging software called Statistical Parametric Mapping version 12 (SPM12, RRID:SCR_007037; University College London, London, United Kingdom). Once manually oriented, the images were co-registered with the Piglet Brain Atlas (Fil et al., 2021b) to ensure proper alignment. Individual pig images were then non-linearly registered to the Piglet Brain Atlas in FSL by using the FNIRT command and inverse warp. Absolute volumes were calculated for 27 different regions of interest (ROI) with “fslmaths” commands in FSL. Relative volumes were also calculated using the following equation for total brain volume (% TBV): (region of interest absolute volume)/(total brain absolute volume) × 100, for each subject. Pig-specific tissue probability maps were utilized to obtain values for gray matter, white matter, and cerebral spinal fluid (CSF) with the “Segment” option in SPM12.

#### Diffusion Tensor Imaging Acquisition and Analysis

A diffusion-weighted echo planar imaging (DW-EPI) sequence was used to assess microscopic water movement within the brain providing information on microstructural development. The following parameters were used: repetition time = 5,100 ms; echo time = 70 ms; GRAPPA accelerated by a factor of 2 in the phase encode direction; diffusion weightings = 1,000 and 2,000 s/mm across 30 directions. Fifty slices with a 1.6 mm thickness were collected with a matrix size of 100 × 100 for a final voxel size of 1.6 mm isotropic. Utilizing the diffusion toolbox in FSL, outcomes were produced for axial diffusivity (AD), mean diffusivity (MD), radial diffusivity (RD), and fractional anisotropy (FA) as previously described (Radlowski et al., 2014). Values were obtained for the following regions of interest (ROI): cerebellum, corpus callosum, left and right caudate, left and right hippocampi, left and right sides of the brain, thalamus, white matter, and overall mask. To transfer each ROI into DTI space, masks for each ROI underwent non-linear transformation into MPRAGE space for each pig and then a linear transform was applied. A FA threshold of 0.5 was applied to ensure only inclusion of white matter in each ROI.

### Statistical Analysis

Both MRI and behavioral outcomes were analyzed by two-way analysis of variance (ANOVA) with a Dunnett’s adjustment using the MIXED procedure of SAS (RRID:SCR_008567; version 9.3; SAS Inst. Inc., Cary, NC, United States), with the exception of recognition memory. To assess recognition memory, the recognition index was compared to a chance performance value of 0.50 using a one-sample *t*-test. Main interaction effects were assessed by comparing all four treatments to each other as well as pairwise comparisons using the Dunnett’s procedure to assess differences between the CON and all other groups. All statistical methods included cohort as a blocking factor, with litter of origin nested within cohort. Transformation was applied to variables where the homogeneity assumption was violated to generate *P*-values, but raw (non-transformed) means and standard error are displayed in all tables and figures. The level of significance was set at *P* < 0.05. Data were expressed both as interaction means (i.e., pigs assigned to each of the 4 experimental treatment groups) or as main effect means when collapsing across levels of the opposite factor.

## Results

### Neuroimaging Outcomes

A total of 52 pigs successfully underwent MRI procedures. During processing for absolute volumes, issues arose for images from one pig, causing data to be unusable. Thus, final sample sizes of 13 CON, 13 BI, 13 FL, and 12 FLBI pigs were used for absolute and relative volume analysis. During diffusion tensor imaging (DTI) processing, signal-to-noise ratio was high resulting in corrupted images. The data for 3 pigs (2 FL and 1 FLBI) were removed from the final data set. Two pigs were identified as outliers (1 FL and 1 BI) and were also removed from the DTI final data set. Thus, final sample sizes of 13 CON, 12 BI, 10 FL, and 11 FLBI were used for DTI analysis.

#### Absolute and Relative Volumes

**FIGURE 1:**
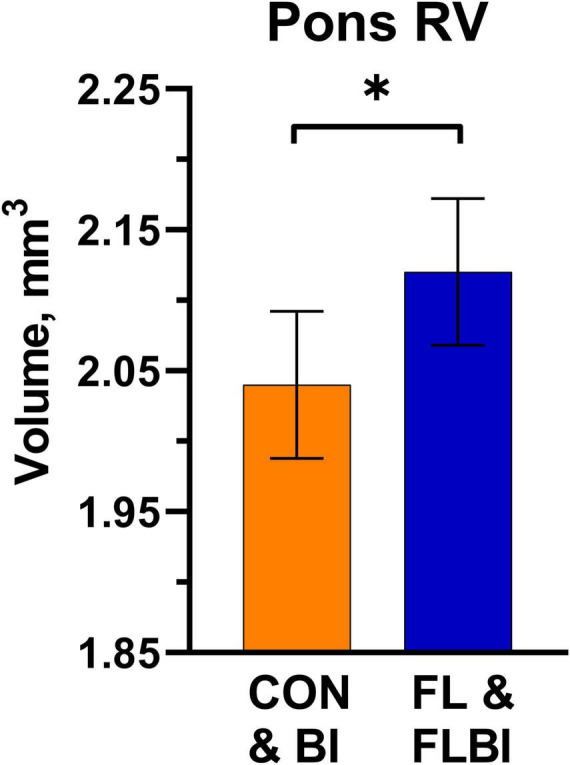
Pons relative volume results. Differences (*P* = 0.046) in relative volume for the Pons brain region were observed between the groups supplemented with 2′-FL (FL and FLBI) compared with those that did not receive 2′-FL supplementation (CON and BI). Groups receiving 2′-FL had larger relative volumes for this brain region. *Asterisk denotes a significant difference between group means (*P* < 0.05). CON, group receiving control diet; FL, group receiving control diet + 1.0 g/L 2′fucosyllactose; BI, group receiving control diet + 10^9^ CFU Bi-26/day; FLBI, group receiving control diet + 1.0 g/L 2′fucosyllactose + 10^9^ CFU Bi-26/day.

To assess main effects of prebiotic supplementation, treatments were grouped by FL and FLBI vs. CON and BI. A main effect was observed for relative volume in the pons region (*P* = 0.046). Pigs that received the prebiotic were found to have larger relative volumes for the pons (Figure 1 and Supplementary Table 1). No differences were observed for absolute brain volume between pigs provided the test diet and pigs on the CON diet (Supplementary Table 2). To assess main effects of probiotic supplementation, treatments were then grouped by BI and FLBI vs. CON and FL. Differences (*P* < 0.05) in absolute brain volume were observed for the following brain regions: corpus callosum, left and right internal capsules, left and right putamen-globus pallidus, left caudate, left cortex, lateral ventricles, and medulla (Table 1). Differences in relative volume were also observed for the left and right putamen-globus pallidus (*P* < 0.03) (Supplementary Table 3). Pigs that were not supplemented with the probiotic (CON and FL) had larger absolute and relative brain volumes for these specified regions of interest compared to those that received probiotic supplementation (BI and FLBI). No interaction effects were observed for either absolute or relative volumes (Supplementary Tables 4, 5).

**TABLE 1:** Absolute brain volumes (mm^3^) of pigs receiving milk replacer treatments differing in probiotic supplementation^1^.

	Main Effect Means		Pooled SEM^2^	Main Effect *P*-value
Region of Interest	CON & FL	BI & FLBI		
*Number of replicate pigs*	*26*	*25*	–	–
Whole brain	60,040	58,951	1,173.6	0.276
White matter	15,640	15,383	283.8	0.506
Gray matter	29,447	29,680	340.6	0.627
Cerebral spinal fluid	2,886	2,827	133.8	0.725
Cerebellum	6,272	6,092	96.3	0.189
Cerebral aqueduct^3^	16	15	0.4	0.529
Corpus callosum	256	243	6.0	**0.015**
Fourth ventricle	19	20	0.8	0.805
Hypothalamus	92	88	1.6	0.063
Lateral ventricle	365	345	9.1	**0.008**
Left caudate	234	225	4.9	**0.032**
Left cortex	17,282	16,545	346.2	**0.023**
Left hippocampus	291	283	4.4	0.146
Left inferior colliculi	72	69	1.0	0.152
Left internal capsule	543	520	9.0	**0.011**
Left olfactory bulb	1,181	1,156	20.4	0.375
Left putamen-globus pallidus	133	125	2.7	**0.002**
Left superior colliculi	169	164	2.2	0.150
Medulla	1,579	1,493	42.7	**0.040**
Midbrain	2,078	2,053	25.0	0.449
Pons	1,264	1,222	24.1	0.113
Right caudate	240	231	5.4	0.069
Right cortex	16,762	16,254	323.8	0.091
Right hippocampus	303	296	5.1	0.210
Right inferior colliculi	73	71	1.2	0.193
Right internal capsule	512	488	9.9	**0.018**
Right olfactory bulb	1,145	1,121	18.8	0.331
Right putamen-globus pallidus	122	113	2.9	**0.003**
Right superior colliculi^3^	176	172	2.5	0.163
Thalamus	1,138	1,107	16.3	0.067
Third ventricle^3^	23	21	1.2	0.138

*^1^Data presented are least squares means and P-values from mixed model 2-way ANOVA. Bolded values denote significance (P < 0.05).*

*^2^Abbreviations: CON, control without supplementation; FL, 2′-FL supplementation; BI, Bi-26 administration; FLBI, 2′-FL supplementation and Bi-26 administration; SEM, standard error of mean.*

*^3^Data transformation was necessary due to a violation of the homogeneity of variance assumption.*

#### Diffusion Tensor Imaging

**FIGURE 2:**
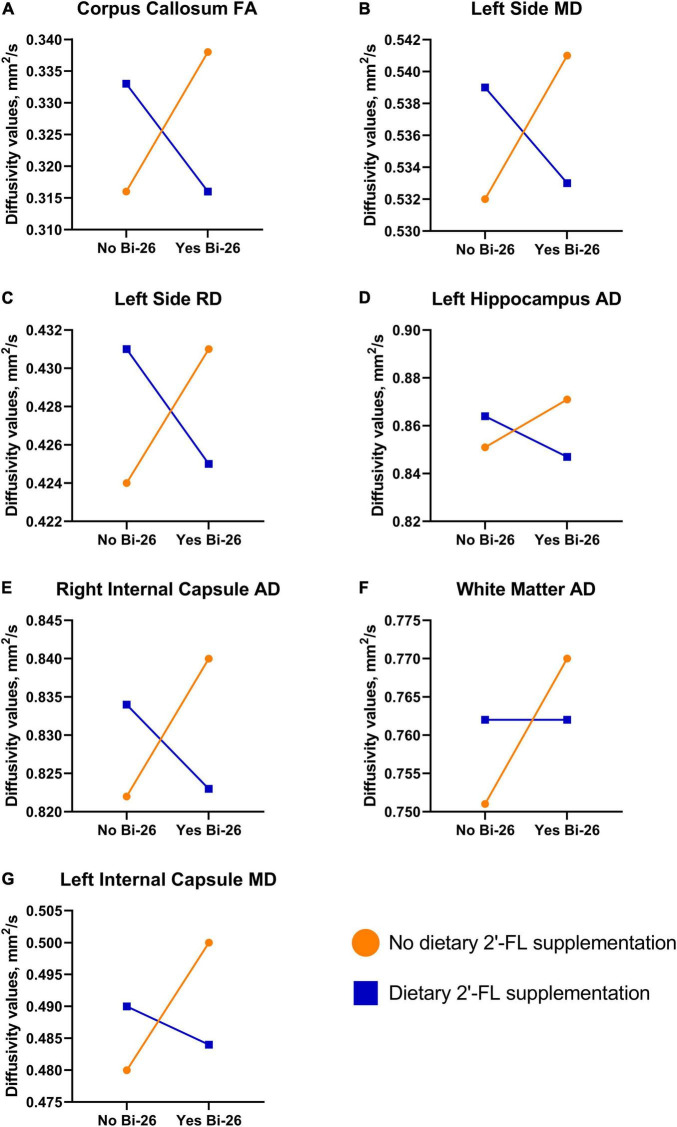
Diffusion tensor imaging interaction results. Various interactive effects were observed between the different treatment groups. **(A–E)** A similar trend was observed for FA in the corpus callosum (*P* = 0.030), MD and RD in the left side of the brain (*P* < 0.019), AD in the left hippocampus (*P* = 0.025), and AD in the right internal capsule (*P* = 0.042). For these regions supplementation with 2′-FL and Bi-26 separately increased values, but these effects were negated when the test articles were supplemented in tandem. **(F,G)** For AD in overall white matter (*P* = 0.039) and MD in the left internal capsule (*P* = 0.006), a pair-wise comparison indicated that Bi-26 supplementation alone led to increased values. AD, axial diffusivity; Bi-26, *Bifidobacterium longum* subsp. *infantis-26*; FA, fractional anisotropy; MD, mean diffusivity; RD, relative diffusivity; 2′-FL, 2′-fucosyllactose.

Several main and interaction effects were observed between the different treatment conditions (Figure 2 and Supplementary Tables 6–17). A main effect was observed for axial diffusivity in the left internal capsule (*P* = 0.025) and overall white matter (*P* = 0.047), where pigs receiving the probiotic (CON and BI) had larger AD values compared to FL and FLBI (Supplementary Table 7). Furthermore, BI pigs had larger AD values in overall white matter compared to CON pigs (*P* = 0.039). Additionally, a main interaction effect was observed for axial diffusivity in the left hippocampus (*P* = 0.025) and right internal capsule (*P* = 0.042) (Supplementary Table 8). Main interaction effects for both mean and radial diffusivity were observed in the left side of the brain (*P* < 0.019) (Supplementary Tables 11, 14). For the left internal capsule, BI pigs displayed larger MD values compared to CON pigs (*P* = 0.006). A main interaction effect was observed for fractional anisotropy in the corpus callosum (*P* = 0.030) (Supplementary Table 17).

### Behavioral Outcomes

Behavioral outcomes from the NOR task were measured to assess the effects of 2′-FL and Bi-26 both alone and in combination on object recognition memory and object exploratory behaviors. A one-sample *t*-test comparing the recognition index with a null performance of 0.50 was conducted to determine whether pigs in four treatment groups demonstrated novelty preference. Overall, none of the four treatment groups demonstrated a novelty preference, as indicated by no recognition index values being higher than 0.50 (*P* > 0.05; Table 2).

**TABLE 2:** Recognition memory on the NOR task with a 48-h delay^1^.

Diet	*n*	Mean	SEM	*P*-Value^2^
***No Bi-26 Supplementation***				
CON	12	0.47	0.037	0.784
FL	13	0.52	0.032	0.258
***Bi-26 Supplementation***				
BI	12	0.54	0.062	0.260
FLBI	10	0.53	0.084	0.371

*^1^Abbreviations: SEM, standard error of mean; BI-26, Bifidobacterium longum subsp. infants; CON, group receiving control diet; FL, group receiving control diet + 1.0 g/L 2′fucosyllactose; BI, group receiving control diet + 10^9^ CFU Bi-26/day; FLBI, group receiving control diet + 1.0 g/L 2′fucosyllactose + 10^9^ CFU Bi-26/day.*

*^2^ P-value derived from one-tailed t-test for a recognition index above 0.50.*

**FIGURE 3:**
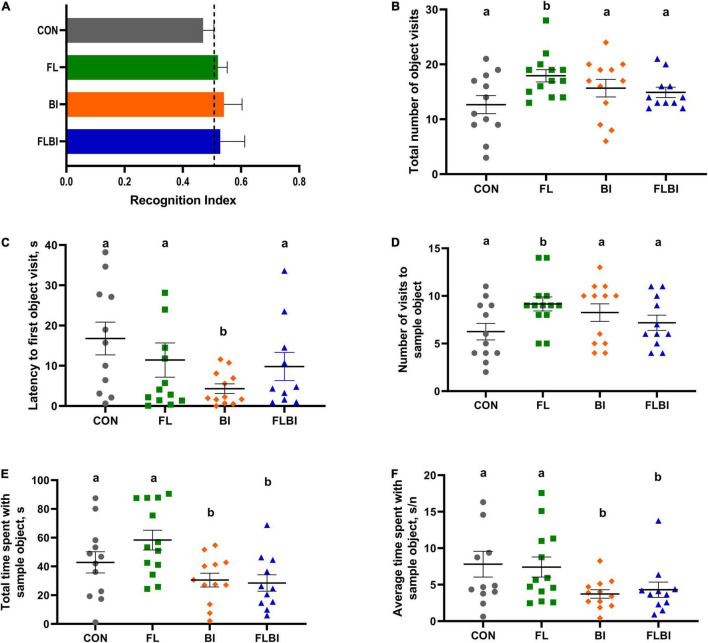
Outcomes from the novel object recognition task. **(A)** Recognition index during the test trial was used as a measure of recognition memory. The dashed line at 0.50 indicates the chance performance value. No treatment groups exhibited recognition memory (*P* > 0.05). **(B–F)** Object exploratory behaviors were measured. **(B,C)** Total number of object visits and the latency to the first event of object exploration were reported. The FL group exhibited a higher number of object visits (*P* = 0.023), and the BI group showed the shorter latency to the first object visit (*P* = 0.023). These effects were not observed in the FLBI group (*P* > 0.05). **(D–F)** Object exploratory behaviors derived from exploring the familiar object were reported. The FL group had a higher number of visits (*P* = 0.022), but this effect disappeared in the FLBI group (*P* > 0.05). The BI and FLBI groups combined (i.e., the pigs that received Bi-26 supplementation) spent less time exploring the sample object in total (*P* = 0.002) and in average per visit (*P* = 0.005). Superscript letters (ab) denote differences (*P* < 0.05) between the treatment means (i.e., means without a common superscript letter are significantly different). CON, group receiving control diet; FL, group receiving control diet + 1.0 g/L 2′fucosyllactose; BI, group receiving control diet + 10^9^ CFU Bi-26/day; FLBI, group receiving control diet + 1.0 g/L 2′fucosyllactose + 10^9^ CFU Bi-26/day.

Pig performance during the test trial of the NOR task after 2-way ANOVA can be found in Tables 3–5 and Figure 3. Table 3 contains the exploratory behaviors regardless of the novelty of the object (i.e., both novel and familiar objects), whereas Tables 4, 5 summarize the exploratory behaviors on the novel and sample (i.e., familiar) objects, respectively. An interaction effect (*P* = 0.023) was noted for the number of objects visits, and a pairwise comparison of interaction means resulted in statistical differentiation between treatment groups. The FL group resulted in a higher number of object visits compared with the CON group. Another interaction effect (*P* = 0.023) was observed for the latency to the first object visit. Pairwise comparison of interaction means exhibited shorter latency in the BI group. No main or interactive effects (*P* > 0.05) for prebiotic and probiotic supplementations were observed for any exploratory behavior measurements involving engagement with the novel object during the test trial. Lastly, regarding engagement with the sample (i.e., familiar) object, pigs that received the Bi-26 supplementation spent less (*P* = 0.002) time investigating the sample object overall (total seconds), which was reinforced by those pigs maintaining a shorter (*P* = 0.005) engagement time each time they visited the sample object (s/visit). Both of these were main effects of Bi-26 supplementation, which indicates that this effect of Bi-26 was not 2′-FL supplementation dependent. An interaction effect (*P* = 0.022) was observed for number of sample object visits, and pairwise comparison of interaction means, which resulted in a more frequent number of sample object visits in the FL group, compared with the CON group.

**TABLE 3:** Exploratory behavior of both objects during the test trial of the NOR task^1^.

Measurements	RI^3^	Total object visit time, s	Number of all object visits, n	Mean object visit time, s/visit	Latency to first object visit, s^3^	Latency to last object visit, s^3^
***Effect of 2*′*-FL***						
Control	0.51	88.0	14.3	6.0	10.6	272.3
Test	0.52	100.8	16.4	6.2	12.3	275.3
SEM	0.039	11.02	1.12	0.77	2.69	7.80
*Effect of Bi-26*						
No Bi-26	0.50	106.7	15.4	7.1	14.1	265.9
Bi-26	0.54	82.1	15.4	5.1	8.7	281.7
SEM	0.040	11.31	1.13	0.77	2.69	7.87
***Interaction means***						
CON	0.47	88.9	12.8^a^	6.8	16.8^a^	255.0
FL	0.52	124.5	18.0^b^	7.5	11.4^a^	276.8
BI	0.54	87.1	15.9^a^	5.2	4.3^b^	289.6
FLBI	0.53	77.1	14.9^a^	5.0	13.2^a^	273.7
SEM	0.058	16.33	1.51	1.11	3.89	10.72
***P-value^2^***						
2′-FL main effect	0.924	0.415	0.118	0.830	0.987	0.933
Bi-26 main effect	0.338	0.121	0.982	0.061	0.125	0.143
Interaction	0.392	0.150	**0.023**	0.702	**0.023**	0.139

*^ab^Superscript letters within a column denote differences between interaction means as derived from the Dunnett’s test (P < 0.05).*

*^1^Abbreviations: RI, recognition index; 2′-FL, 2-fucosyllactose; SEM, standard error of mean; Bi-26, Bifidobacterium longum subsp. infantis; CON, group receiving control diet; FL, group receiving control diet + 1.0 g/L 2′fucosyllactose; BI, group receiving control diet + 10^9^ CFU Bi-26/day; FLBI, group receiving control diet + 1.0 g/L 2′fucosyllactose + 10^9^ CFU Bi-26/day.*

*^2^ P-value derived from two-way ANOVA for the main effects and the interaction. Bolded values denote significance (P < 0.05).*

*^3^Data transformation has been completed due to a violation of the homogeneity of variance assumption.*

**TABLE 4:** Exploratory behavior of the novel object during the test trial of the NOR task^1^.

Measurements	Total novel object visit time, s	Number of novel object visits, n	Mean novel object visit time, s/visit	Latency to first novel object visit, s	Latency to last novel object visit, s
***Effect of 2*′*-FL***					
Control	51.3	7.1	7.1	27.0	250.0
Test	57.3	8.3	6.7	26.0	244.7
SEM	7.69	0.90	0.94	6.22	12.55
***Effect of Bi-26***					
No Bi-26	56.1	7.7	7.6	28.2	232.9
Bi-26	52.6	7.6	6.2	24.9	261.4
SEM	7.83	0.91	0.95	6.22	12.29
***Interaction means***					
CON	46.0	6.6	7.2	35.0	229.0
FL	66.1	8.9	7.9	21.3	236.8
BI	56.6	7.7	7.0	19.1	270.2
FLBI	48.5	7.6	5.5	30.7	252.7
SEM	11.31	1.11	1.38	8.80	16.85
***P-value^2^***					
2′-FL main effect	0.583	0.182	0.780	0.940	0.761
Bi-26 main effect	0.749	0.879	0.318	0.705	0.060
Interaction	0.201	0.155	0.396	0.149	0.395

*^1^Abbreviations: 2′-FL, 2-fucosyllactose; SEM, standard error of mean; Bi-26, Bifidobacterium longum subsp. infantis; CON, group receiving control diet; FL, group receiving control diet + 1.0 g/L 2′fucosyllactose; BI, group receiving control diet + 10^9^ CFU Bi-26/day; FLBI, group receiving control diet + 1.0 g/L 2′fucosyllactose + 10^9^ CFU Bi-26/day.*

*^2^P-value derived from repeated-measures ANOVA for the main effects and the interaction.*

**TABLE 5:** Exploratory behavior of the sample (familiar) object during the test trial of the NOR task^1^.

Measurements	Total sample object visit time, s	Number of sample object visits, n	Mean sample object visit time, s/visit^3^	Latency to first sample object visit, s^3^	Latency to last sample object visit, s
***Effect of 2*′*-FL***					
Control	36.7	7.3	5.7	30.3	251.9
Test	43.5	8.2	5.9	25.1	254.3
SEM	4.65	0.59	1.03	6.23	9.72
***Effect of Bi-26***					
No Bi-26	50.7	7.7	7.7	31.2	245.2
Bi-26	29.5	7.7	4.0	24.1	260.9
SEM	4.70	0.60	1.04	6.23	9.91
***Interaction means***					
CON	42.9	6.3^a^	7.8	36.5	236.2
FL	58.5	9.2^b^	7.5	26.0	254.2
BI	30.4	8.3^a^	3.6	24.0	267.6
FLBI	28.6	7.2^a^	4.3	24.2	254.3
SEM	6.74	0.87	1.42	9.01	14.31
***P-value^2^***					
2′-FL main effect	0.289	0.278	0.786	0.526	0.863
Bi-26 main effect	**0.002**	0.987	**0.005**	0.136	0.258
Interaction	0.179	**0.022**	0.799	0.213	0.260

*^ab^Superscript letters within a column denote differences between interaction means as derived from the Dunnett’s test (P < 0.05).*

*^1^Abbreviations: 2′-FL, 2-fucosyllactose; SEM, standard error of mean; Bi-26, Bifidobacterium longum subsp. infantis; CON, group receiving control diet; FL, group receiving control diet + 1.0 g/L 2′fucosyllactose; BI, group receiving control diet + 10^9^ CFU Bi-26/day; FLBI, group receiving control diet + 1.0 g/L 2′fucosyllactose + 10^9^ CFU Bi-26/day.*

*^2^P-value derived from repeated-measures ANOVA for the main effects and the interaction. Bolded values denote significance (P < 0.05).*

*^3^Data transformation has been completed due to a violation of the homogeneity of variance assumption.*

## Discussion

In the present study, the effects of 2′-FL and B-26 individually and together on brain structural and behavioral development were assessed in young pigs. Results on growth performance and intestinal health outcomes of this study were previously reported (Daniels et al., 2022).

### Magnetic Resonance Imaging

To understand the implications that 2′-FL and Bi-26 had on structural brain development, anatomical and DTI scans were performed on pigs. Absolute and relative volumes were conducted on the whole brain, white matter, gray matter, cerebral spinal fluid (CSF), and on an additional 27 different regions of interest. Differences between larger regions were not observed indicating similar trajectory in overall brain growth between the different treatment conditions.

Additionally, values for absolute and relative volumes are relatively consistent with expected volumes for the young pig (Fil et al., 2021b), suggesting that supplementation with either 2′-FL or Bi-26 was safe and well tolerated. Regarding relative volumes, the pons region was observed to be larger in pigs supplemented with 2′-FL (FL and FLBI) relative to overall brain growth, compared to those that did not receive the prebiotic (CON and BI). However, no other effects were observed indicating minimal macrostructure changes associated with 2′-FL supplementation. Previous research by Fleming et al. (2020) indicated more structural outcomes associated with 2′-FL supplementation, as well as interaction effects where 2′-FL was observed to lead to larger relative volumes in the pons region. However, this effect was only seen with the inclusion of bovine milk oligosaccharides as an interaction.

Supplementation with Bi-26 was associated with a larger array of structural outcomes. Although overall white matter concentrations did not differ, several white matter-associated regions were impacted such as the corpus callosum and internal capsules. Interestingly, the addition of Bi-26 (BI and FLBI) was associated with smaller absolute volumes in these regions compared to those that did not receive the probiotic (CON and FL). Similarly, differences in overall gray matter concentrations were not observed but in gray matter structures such as the left and right putamen globus-pallidus, which make up the lentiform nuclei, smaller values were observed in both absolute and relative volume between the Bi-26 supplementation groups. Explicitly, for some regions, smaller absolute volume with Bi-26 supplementation was observed only for the left side of the brain—left cortex and left caudate nucleus. Together the caudate nuclei, putamen, and globus pallidus form the corpus striatum, which is an important structure for the control of voluntary movement (Carpenter, 1984). Additionally, smaller absolute volume in the pigs supplemented with Bi-26 was observed in the medulla, a major route for sensory and motor tracts, as well as the lateral ventricles, sites of major CSF production in the brain. Overall, there is evidence that Bi-26 supplementation affected regions associated with movement and motor skills as well as several white matter regions. It is unclear as to what the direct implications are of these findings as this is the first study, to our knowledge, to assess how Bi-26 supplementation relates to structural brain development.

Diffusion tensor imaging (DTI) was used to understand the microstructural organization of the brain. Four different components are obtained through DTI to determine white matter anatomy: fractional anisotropy (FA) assesses the degree to which diffusion occurs in one direction, axial diffusivity (AD) measures diffusion rate parallel to main fibers, radial diffusivity (RD) measures diffusion rate perpendicular to main fibers, and mean diffusivity (MD) assesses the average diffusion rate (Alexander et al., 2007). In the present study, several interactions and main effects were observed between the different treatment conditions. Supplementation with 2FL or Bi-26 separately seemed to increase FA in the corpus callosum, but when supplemented in tandem, the effects were negated, and values were identical to the control group. FA is a more sensitive measure that provides insight into fiber integrity (Alexander et al., 2007). An increased value in FA can indicate maturation in the specified region, as it has been previously documented that FA values tend to increase through infancy into later childhood (McGraw et al., 2002). Additionally, an increase in FA values for the corpus callosum has been previously observed in mice during the first 3 weeks after birth, which is believed to be attributed to axonal pruning and myelination in the rat brain (Bockhorst et al., 2008).

Similar interaction effects were observed for the other diffusivity components in various brain regions. In some instances, a pair-wise comparison indicated Bi-26 supplementation alone led to increased values compared to the control group. Several main effects were present that supported this finding, but it was only observed for axial diffusivity. Previous research has described the difficulty of interpreting AD and RD values due to the complex fiber structure of the brain (Kumar et al., 2012; Winklewski et al., 2018). Typically, with increasing age, AD and RD values seem to decrease in a variety of regions (Kumar et al., 2012). Moreover, it has been observed that with increasing age different patterns are observed. Additionally, FA values are typically accompanied by corresponding decreases or increases in RD or AD values that vary based on the specific brain region (Bennett et al., 2010). These various patterns can provide an understanding of the different trajectories of maturity throughout the brain. The first 3–11 months of infancy are marked by large changes in growth of axons, axon diameter, and myelination which can affect the macrostructure and microstructure of the brain (Deoni et al., 2011). Changes and fluctuations, especially in diffusivity values, may be an indication of brain maturation (Ouyang et al., 2019). Since the 4-week time-point is noted as being the time for most rapid brain growth in the pig, it is possible that this is a critical moment in maturation and fluctuations should be expected (Conrad et al., 2012). However, many of the DTI studies that have been mentioned were completed at multiple age time points following the same subjects longitudinally, which is a limitation in the current study. Additionally, the age groups that were assessed in many human DTI studies are older (Bennett et al., 2010; Kumar et al., 2012) than the pediatric group that was targeted in this study using a 4-week-old pig. Due to these limitations, it is difficult to interpret the current findings. Overall, brain microstructure was affected by 2′-FL and Bi-26 supplementation, but further research should be completed to understand the full implications of supplementation on brain microstructure.

Previously it has been found that supplementation with HMOs, specifically 2‘-FL, was linked with improvements in long-term potentiation (LTP) in rodents (Vázquez et al., 2015). LTP is the increased synaptic strength between neurons caused by repeated stimulation and typically affects the memory centers of the brain (Frey and Morris, 1997). Therefore, improvements in LTP have been observed to lead to better performance on learning and memory tasks in rodents (Vázquez et al., 2015). Additionally, synaptogenesis and synaptic refinement are known developmental events that occur as the brain increases in volume throughout the first few years of development (Jena et al., 2020). In the current study, it is possible that changes in LTP, which directly affects synaptic strength, could be the underlying mechanism between the macrostructure and microstructure changes associated with 2‘-FL that were observed. Furthermore, short-chain fatty acids (SCFAs), well-known byproducts of prebiotic fermentation by the gut microbiota, have been suggested to indirectly interact with the brain (Al-Khafaji et al., 2020; Jena et al., 2020; Markowiak-Kopeć and Śliżewska, 2020). Although the mechanisms and implications are not clearly identified, various SCFAs have shown to improve the structure and permeability of the blood-brain-barrier, which is crucial for brain integrity (Persidsky et al., 2006; Braniste et al., 2014; Logsdon et al., 2018). Through these potential modes of action, the various events of neurodevelopment, like synaptogenesis and microstructure organization, could be impacted by 2′-FL and Bi-26 supplementation.

### Behavior

The effects of 2′-FL and Bi-26 on recognition memory and object exploratory behaviors were examined separately and combined using a novelty preference behavioral task for pigs. NOR is a well-established behavioral paradigm in pigs that measures object recognition memory as an indicator of cognition (Fleming et al., 2017). In the present study, no treatment groups exhibited novelty preference or differences in a recognition index. This result suggests no changes in object recognition memory, despite a longer investigation time with the familiar object in total that was observed in the pigs that did not receive Bi-26 supplementation (i.e., CON and FL groups combined). These pigs also exhibited a longer time spent with the familiar object on average per visit, and more visits to the familiar object made by FL group pigs were also observed. The recognition index is closely related to the time spent with each object, as it is a proportion of time spent investigating the novel object to time spent with both familiar and novel objects combined. Thus, spending more time with the familiar object may result in a lower recognition index. However, the main effects of Bi-26 observed in the total time and averaged time spent with the familiar object did not appear to have driven a similar effect on the recognition index. Collectively, dietary supplementation of Bi-26 demonstrated a minor difference in object exploratory behaviors, which did not translate into any changes in object recognition memory.

The beneficial effects of bifidobacteria strains on cognitive capabilities have been reported in rodent studies that used the NOR task (Allen et al., 2016; Savignac et al., 2015; Talani et al., 2020). Adult rats that received a mixture of different strains of bifidobacteria exhibited higher recognition index in the NOR task, suggesting an improvement in cognitive performance compared to the control group rats (Talani et al., 2020). Also, the adult mice that received *B. longum* 1714 strains demonstrated object recognition memory earlier than other groups, which was suggestive of an improvement in object recognition memory (Savignac et al., 2015). The same study also tested other aspects of cognition and observed enhanced performance when using the Barnes maze in the mice that received *B. longum* 1714 compared with the control group (Savignac et al., 2015). Similar results were also observed in a human study investigating effects of *B. longum* 1714 supplementation on cognitive performance. Allen et al. (2016) observed improved performance on the paired associate learning test in healthy male adult volunteers, suggesting that the *B. longum* 1714 supplementation enhanced the visuospatial memory.

Despite the beneficial effects of Bi-26 and 2′-FL supplementations highlighted in the literature, the discrepancies between previous findings and the results from our study may be explained by the time-point at which the animals were tested for cognitive performance. The current study incorporated the cognitive behavioral task at a single time-point and the testing was completed at a relatively young age. Specifically, pigs in the present study were tested for the NOR task at PND 31, which resembles infancy in humans since 1 week in the brain growth of pigs approximates 1 month of brain growth in infants (Conrad and Johnson, 2015). However, much literature supporting the positive effects of *B. longum* and 2′-FL on cognitive development include the cognitive testing at an older age. As such, 2′-FL has been suggested to have temporal window-specific beneficial effects on cognitive development, which was not observed at a young age, but only became apparent at a later time-point in life. Thus, having a longitudinal study including cognitive testing at an older age may further provide insights into the long-lasting effects of Bi-26 and 2′-FL on cognitive functioning.

Another interesting result observed from the current study is that the BI group exhibited a shorter latency measure to the first object visit, a finding that was not present in the FLBI group. The differences in the latency measure in the NOR task due to early-life nutritional changes have been observed previously (Joung et al., 2020). It was suspected that the latency measures may be more closely related to anxiety-related behaviors, rather than object recognition memory, and that the shorter latency to the first object encounter may suggest a reduced anxiety level. Supporting this hypothesis, potential anxiolytic effects of various strains of probiotics have been increasingly gaining popularity for therapeutic potentials (c.f., Foster and McVey Neufeld, 2013; Slyepchenko et al., 2014; Luna and Foster, 2015). Specifically, the potential anxiolytic effects of *Bifidobacterium* strains have been investigated in many animal and clinical studies. *B. longum* has demonstrated a reduction in anxiety-like behaviors in mice that had an increase in the gastrointestinal inflammation-dependent anxiety level (Bercik et al., 2010, 2011). Similarly, *B. longum* NCC3001 elicited decreased anxiety not only in healthy mice, but also in mice with dextran sodium sulfate-induced colitis mediated by the vagus nerve (Bercik et al., 2011). A probiotic blend of *B. longum* R0175 and *L. helveticus* R0052 also exhibited anxiolytic effects on the marble burying test in rats (Messaoudi et al., 2011a) and the hospital anxiety and depression scale in humans (Messaoudi et al., 2011b). Another clinical study demonstrated that *B. infantis* M-63 improved mental health in flood-affected individuals with irritable bowel syndrome (Ma et al., 2019). Although the synbiotic effects of 2′-FL and Bi-26 on anxiety are not well-established, possible underlying mechanisms of gut microbiota have been explored in growing body of literature in recent years. Bifidobacteria strains demonstrated attenuated pro-inflammation responses in rodents (Desbonnet et al., 2008; Messaoudi et al., 2011a), and restored a balance of anti-inflammatory and pro-inflammatory cytokines in irritable bowel syndrome patients (O’Mahony et al., 2005). Also, *B. infantis* increased plasma concentration of tryptophan, a precursor for serotonin, which plays a crucial role in emotion processing and mood disorders such as depression and anxiety (Desbonnet et al., 2008, 2010). Thus, potential anxiolytic effects of *B. infantis* supplementation during early-life may have multiple plausible mediators, such as the pro-inflammatory cytokines and brain monoamines. However, given the lack of a proper validation for the latency measures from the NOR task in pigs to assess anxiety-related behavior, it is important to note that the interpretation of reduced anxiety level in the BI group from the present study cannot be conclusive at this point. Also, the majority of findings are focusing on the *B. longum* strain specifically, and the anxiolytic effect of *B. infantis* was not studied as much. Lastly, the disappearance of the effect of Bi-26 in the latency measure in the FLBI group suggests that the potential anxiolytic effect of Bi-26 may not translate to the synergistic effects of 2′-FL and Bi-26. This is consistent with the effect of 2′-FL on the presence of *B. infantis* in the ascending colon or rectum not translating to a synbiotic effect when co-administered with Bi-26 (Daniels et al., 2022). Overall, further research is warranted to study potential anxiolytic effects of probiotics, especially *B. infantis*, and synbiotics with a properly validated behavioral model for anxiety in pigs.

## Conclusion

Normative development of the gut-brain-axis is critical for not only overall health but also brain structural and functional development. The relationship between HMOs and the gut microbiota plays an essential role in the gut-brain-axis, and various potentially beneficial effects of prebiotics and probiotics, specifically 2′-FL and *Bifidobacterium* species, have been investigated in the literature. As the brain is experiencing rapid growth and maturation, these findings imply potential alterations in the structure and functions of the brain that may arise from the supplementation of 2‘-FL and Bi-26. However, further research is warranted to investigate the underlying mechanisms of their effects on potential anxiolytic effects and organization of brain fiber structure and how synbiotic supplementation may differ from prebiotic and probiotic outcomes observed separately.

## Data Availability Statement

The datasets presented in this article are not readily available because commercial products were tested. Requests to access the datasets should be directed to RD, rdilger2@illinois.edu.

## Ethics Statement

The animal study was reviewed and approved by University of Illinois at Urbana-Champaign Institutional Animal Care and Use Committee.

## Author Contributions

SD and RD obtained funding for the research. SD, RD, JH, HJ, AO, and RM designed the study. LS and SJ participated in sample collection, data analysis, and prepared the manuscript. All authors have read and approved the manuscript.

## Conflict of Interest

JH, HJ, and AO are employees of IFF while RM was employed by IFF at the time of study. The remaining authors declare that the research was conducted in the absence of any commercial or financial relationships that could be construed as a potential conflict of interest.

## Publisher’s Note

All claims expressed in this article are solely those of the authors and do not necessarily represent those of their affiliated organizations, or those of the publisher, the editors and the reviewers. Any product that may be evaluated in this article, or claim that may be made by its manufacturer, is not guaranteed or endorsed by the publisher.
