# Supplementary material for: Influence of 2′-Fucosyllactose and Bifidobacterium longum Subspecies infantis Supplementation on Cognitive and Structural Brain Development in Young Pigs
Source: Front Neurosci. 2022 Apr 25;16:860368. doi: 10.3389/fnins.2022.860368 (PMC9081927; doi:10.3389/fnins.2022.860368)
Supplement: Supplementary file 1 [file Table_1.docx]

| Supplemental Table 1. Relative brain volumes (% of total brain volume) of pigs receiving milk replacer treatments differing in prebiotic supplementation^1^ | | | | |
| --- | --- | --- | --- | --- |
|  | **Main Effect Means** | |  | **Main Effect** |
| Region of Interest | **CON & BI** | **FL & FLBI** | **Pooled SEM^2^** | ***P*-value** |
| *Number of replicate pigs* | *26* | *25* | **-** | **-** |
| Gray matter | 49.74 | 49.72 | 0.876 | 0.982 |
| White matter | 25.75 | 26.36 | 0.422 | 0.266 |
| Cerebral spinal fluid^3^ | 4.72 | 4.97 | 0.285 | 0.622 |
| Cerebellum | 10.41 | 10.40 | 0.219 | 0.966 |
| Cerebral aqueduct^3^ | 0.03 | 0.03 | 0.001 | 0.607 |
| Corpus callosum | 0.42 | 0.42 | 0.010 | 0.585 |
| Fourth ventricle^3^ | 0.03 | 0.04 | 0.002 | 0.130 |
| Hypothalamus | 0.15 | 0.15 | 0.002 | 0.616 |
| Lateral ventricle^3^ | 0.60 | 0.60 | 0.016 | 0.870 |
| Left caudate | 0.39 | 0.39 | 0.010 | 0.788 |
| Left cortex | 28.12 | 28.65 | 0.580 | 0.164 |
| Left hippocampus | 0.49 | 0.48 | 0.008 | 0.666 |
| Left inferior colliculi^3^ | 0.12 | 0.12 | 0.002 | 0.483 |
| Left internal capsule^3^ | 0.90 | 0.89 | 0.021 | 0.815 |
| Left olfactory bulb^3^ | 1.95 | 1.98 | 0.035 | 0.527 |
| Left putamen-globus pallidus | 0.22 | 0.22 | 0.006 | 0.666 |
| Left superior colliculi^3^ | 0.28 | 0.28 | 0.005 | 0.498 |
| Medulla^3^ | 2.53 | 2.65 | 0.088 | 0.079 |
| Midbrain | 3.47 | 3.46 | 0.062 | 0.951 |
| Pons | 2.04 | 2.12 | 0.052 | 0.046 |
| Right caudate | 0.40 | 0.40 | 0.010 | 0.847 |
| Right cortex | 27.60 | 27.66 | 0.435 | 0.840 |
| Right hippocampus^3^ | 0.51 | 0.50 | 0.010 | 0.126 |
| Right inferior colliculi^3^ | 0.12 | 0.12 | 0.002 | 0.772 |
| Right internal capsule | 0.84 | 0.84 | 0.022 | 0.967 |
| Right olfactory bulb^3^ | 1.91 | 1.90 | 0.032 | 0.988 |
| Right putamen-globus pallidus | 0.20 | 0.20 | 0.005 | 0.896 |
| Right superior colliculi^3^ | 0.30 | 0.29 | 0.005 | 0.175 |
| Thalamus | 1.90 | 1.90 | 0.038 | 0.931 |
| Third ventricle^3^ | 0.04 | 0.04 | 0.002 | 0.251 |
| ^1^Data presented are least squares means and *P*-values from mixed model 2-way ANOVA.  ^2^Abbreviations: CON, control without supplementation; FL, 2’-FL supplementation; BI, Bi-26 administration; FLBI, 2’-FL supplementation and Bi-26 administration; SEM, standard error of mean.  ^3^Data transformation was necessary due to a violation of the homogeneity of variance assumption. | | | | |

| Supplemental Table 2. Absolute brain volumes (mm^3^) of pigs receiving milk replacer treatments differing in prebiotic supplementation^1^ | | | | |
| --- | --- | --- | --- | --- |
|  | **Main Effect Means** | |  | **Main Effect** |
| Region of Interest | **CON & BI** | **FL & FLBI** | **Pooled SEM^2^** | ***P*-value** |
| *Number of replicate pigs* | *26* | *25* | **-** | **-** |
| Whole Brain | 59972 | 59019 | 1174.6 | 0.347 |
| Gray matter | 29763 | 29363 | 340.6 | 0.406 |
| White matter | 15456 | 15567 | 288.7 | 0.779 |
| Cerebral spinal fluid | 2819 | 2895 | 135.7 | 0.654 |
| Cerebellum | 6226 | 6138 | 96.3 | 0.516 |
| Cerebral aqueduct^3^ | 15 | 15 | 0.4 | 0.686 |
| Corpus callosum | 254 | 246 | 6.0 | 0.161 |
| Fourth ventricle | 19 | 20 | 0.8 | 0.331 |
| Hypothalamus | 90 | 90 | 1.6 | 0.964 |
| Lateral ventricle | 359 | 351 | 9.2 | 0.302 |
| Left caudate | 231 | 228 | 4.9 | 0.567 |
| Left cortex | 16913 | 16914 | 346.8 | 0.997 |
| Left hippocampus | 289 | 285 | 4.4 | 0.479 |
| Left inferior colliculi | 71 | 70 | 1.0 | 0.861 |
| Left internal capsule | 536 | 526 | 9.0 | 0.230 |
| Left olfactory bulb | 1169 | 1168 | 20.4 | 0.954 |
| Left putamen-globus pallidus | 130 | 128 | 2.7 | 0.648 |
| Left superior colliculi | 169 | 165 | 2.2 | 0.241 |
| Medulla | 1509 | 1564 | 42.8 | 0.183 |
| Midbrain | 2074 | 2057 | 25.1 | 0.607 |
| Pons | 1233 | 1254 | 24.2 | 0.419 |
| Right caudate | 238 | 233 | 5.4 | 0.310 |
| Right cortex | 16587 | 16428 | 324.4 | 0.591 |
| Right hippocampus | 302 | 297 | 5.1 | 0.336 |
| Right inferior colliculi | 73 | 72 | 1.2 | 0.725 |
| Right internal capsule | 504 | 496 | 10.0 | 0.375 |
| Right olfactory bulb | 1143 | 1123 | 18.8 | 0.441 |
| Right putamen-globus pallidus | 118 | 117 | 2.9 | 0.682 |
| Right superior colliculi^3^ | 176 | 172 | 2.5 | 0.172 |
| Thalamus | 1128 | 1117 | 16.4 | 0.517 |
| Third ventricle^3^ | 22 | 22 | 1.2 | 0.453 |
| ^1^Data presented are least squares means and *P*-values from mixed model 2-way ANOVA.  ^2^Abbreviations: CON, control without supplementation; FL, 2’-FL supplementation; BI, Bi-26 administration; FLBI, 2’-FL supplementation and Bi-26 administration; SEM, standard error of mean.  ^3^Data transformation was necessary due to a violation of the homogeneity of variance assumption. | | | | |

| Supplemental Table 3. Relative brain volumes (% of total brain volume) of pigs receiving milk replacer treatments differing in probiotic supplementation^1^ | | | | |
| --- | --- | --- | --- | --- |
|  | **Main Effect Means** | |  | **Main Effect** |
| Region of Interest | **CON & FL** | **BI & FLBI** | **Pooled SEM^2^** | ***P*-value** |
| *Number of replicate pigs* | *26* | *25* | **-** | **-** |
| Gray matter | 49.12 | 50.34 | 0.874 | 0.233 |
| White matter | 26.04 | 26.07 | 0.415 | 0.968 |
| Cerebral spinal fluid^3^ | 4.84 | 4.85 | 0.282 | 0.918 |
| Cerebellum | 10.47 | 10.35 | 0.218 | 0.569 |
| Cerebral aqueduct^3^ | 0.03 | 0.03 | 0.001 | 0.630 |
| Corpus callosum | 0.43 | 0.41 | 0.010 | 0.058 |
| Fourth ventricle^3^ | 0.04 | 0.03 | 0.002 | 0.529 |
| Hypothalamus | 0.15 | 0.15 | 0.002 | 0.776 |
| Lateral ventricle^3^ | 0.61 | 0.59 | 0.016 | 0.054 |
| Left caudate | 0.39 | 0.38 | 0.009 | 0.193 |
| Left cortex | 28.69 | 28.08 | 0.579 | 0.114 |
| Left hippocampus | 0.49 | 0.48 | 0.008 | 0.185 |
| Left inferior colliculi^3^ | 0.12 | 0.12 | 0.002 | 0.734 |
| Left internal capsule^3^ | 0.91 | 0.88 | 0.021 | 0.080 |
| Left olfactory bulb^3^ | 1.96 | 1.96 | 0.035 | 0.913 |
| Left putamen-globus pallidus | 0.22 | 0.21 | 0.006 | 0.030 |
| Left superior colliculi^3^ | 0.28 | 0.28 | 0.005 | 0.608 |
| Medulla^3^ | 2.64 | 2.54 | 0.088 | 0.147 |
| Midbrain | 3.45 | 3.49 | 0.062 | 0.297 |
| Pons | 2.11 | 2.06 | 0.052 | 0.179 |
| Right caudate | 0.40 | 0.39 | 0.010 | 0.071 |
| Right cortex | 27.69 | 27.57 | 0.435 | 0.679 |
| Right hippocampus^3^ | 0.51 | 0.50 | 0.010 | 0.148 |
| Right inferior colliculi^3^ | 0.12 | 0.12 | 0.002 | 0.743 |
| Right internal capsule | 0.86 | 0.83 | 0.022 | 0.070 |
| Right olfactory bulb^3^ | 1.91 | 1.90 | 0.032 | 0.868 |
| Right putamen-globus pallidus | 0.20 | 0.19 | 0.005 | 0.038 |
| Right superior colliculi^3^ | 0.29 | 0.29 | 0.005 | 0.601 |
| Thalamus | 1.92 | 1.88 | 0.038 | 0.105 |
| Third ventricle^3^ | 0.04 | 0.04 | 0.002 | 0.381 |
| ^1^Data presented are least squares means and *P*-values from mixed model 2-way ANOVA.  ^2^Abbreviations: CON, control without supplementation; FL, 2’-FL supplementation; BI, Bi-26 administration; FLBI, 2’-FL supplementation and Bi-26 administration; SEM, standard error of mean.  ^3^Data transformation was necessary due to a violation of the homogeneity of variance assumption. | | | | |

| Supplemental Table 4. Absolute brain volumes (mm^3^) of pigs assigned to factorial arrangement of prebiotic and probiotic supplementation^1^ | | | | | | |
| --- | --- | --- | --- | --- | --- | --- |
|  | **Interaction Means** | | | |  |  |
| Region of Interest | **CON** | **BI** | **FL** | **FLBI** | **Pooled SEM^2^** | **Interaction *P*-value** |
| *Number of replicate pigs* | *13* | *13* | *13* | *12* | **-** | *-* |
| Whole brain | 60531 | 59413 | 59549 | 58489 | 1397.3 | 0.977 |
| Gray matter | 29203 | 30324 | 29690 | 29036 | 491.2 | 0.069 |
| White matter | 15724 | 15188 | 15557 | 15578 | 407.0 | 0.477 |
| Cerebral spinal fluid | 2960 | 2677 | 2812 | 2978 | 184.4 | 0.182 |
| Cerebellum | 6240 | 6212 | 6303 | 5972 | 138.9 | 0.267 |
| Cerebral aqueduct^3^ | 15 | 16 | 16 | 15 | 0.5 | 0.127 |
| Corpus callosum | 259 | 248 | 254 | 239 | 7.1 | 0.755 |
| Fourth ventricle | 19 | 20 | 20 | 20 | 0.9 | 0.349 |
| Hypothalamus | 92 | 89 | 93 | 88 | 2.3 | 0.728 |
| Lateral ventricle | 368 | 351 | 363 | 339 | 10.8 | 0.623 |
| Left caudate | 232 | 229 | 236 | 220 | 5.9 | 0.165 |
| Left cortex | 17212 | 16614 | 17353 | 16475 | 417.3 | 0.658 |
| Left hippocampus | 292 | 286 | 290 | 280 | 5.9 | 0.689 |
| Left inferior colliculi | 71 | 70 | 72 | 69 | 1.5 | 0.480 |
| Left internal capsule | 545 | 528 | 540 | 512 | 11.2 | 0.545 |
| Left olfactory bulb | 1193 | 1146 | 1169 | 1166 | 30.2 | 0.433 |
| Left putamen-globus pallidus | 132 | 127 | 134 | 123 | 3.3 | 0.282 |
| Left superior colliculi | 170 | 167 | 168 | 162 | 3.2 | 0.715 |
| Medulla | 1550 | 1468 | 1609 | 1518 | 52.3 | 0.918 |
| Midbrain | 2080 | 2068 | 2075 | 2039 | 34.7 | 0.709 |
| Pons | 1251 | 1214 | 1277 | 1231 | 30.9 | 0.853 |
| Right caudate | 240 | 236 | 240 | 226 | 6.6 | 0.372 |
| Right cortex | 16792 | 16382 | 16731 | 16125 | 390.7 | 0.740 |
| Right hippocampus | 306 | 298 | 300 | 293 | 6.7 | 0.954 |
| Right inferior colliculi | 74 | 72 | 73 | 71 | 1.7 | 0.825 |
| Right internal capsule | 515 | 494 | 508 | 483 | 12.4 | 0.857 |
| Right olfactory bulb | 1159 | 1127 | 1132 | 1115 | 27.8 | 0.764 |
| Right putamen-globus pallidus | 122 | 115 | 122 | 112 | 3.5 | 0.591 |
| Right superior colliculi^3^ | 179 | 174 | 174 | 170 | 3.5 | 0.852 |
| Thalamus | 1144 | 1112 | 1133 | 1101 | 20.9 | 0.983 |
| Third ventricle^3^ | 23 | 21 | 23 | 21 | 1.5 | 0.702 |
| ^1^Data presented are least squares means and *P*-values from mixed model 2-way ANOVA.  ^2^Abbreviations: CON, control without supplementation; FL, 2’-FL supplementation; BI, Bi-26 administration; FLBI, 2’-FL supplementation and Bi-26 administration; SEM, standard error of mean.  ^3^Data transformation was necessary due to a violation of the homogeneity of variance assumption. | | | | | | |

| Supplemental Table 5. Relative brain volumes (% of total brain volume) of pigs assigned to factorial arrangement of prebiotic and probiotic supplementation^1^ | | | | | | |
| --- | --- | --- | --- | --- | --- | --- |
|  | **Interaction Means** | | | |  |  |
| Region of Interest | **CON** | **BI** | **FL** | **FLBI** | **Pooled SEM^2^** | **Interaction *P*-value** |
| *Number of replicate pigs* | *13* | *13* | *13* | *12* | **-** | *-* |
| Gray matter | 48.41 | 51.07 | 49.84 | 49.60 | 1.160 | 0.158 |
| White matter | 25.96 | 25.53 | 26.13 | 26.60 | 0.583 | 0.414 |
| Cerebral spinal fluid^3^ | 4.91 | 4.54 | 4.78 | 5.16 | 0.367 | 0.283 |
| Cerebellum | 10.34 | 10.48 | 10.59 | 10.21 | 0.268 | 0.208 |
| Cerebral aqueduct^3^ | 0.02 | 0.03 | 0.03 | 0.03 | 0.001 | 0.164 |
| Corpus callosum | 0.43 | 0.42 | 0.43 | 0.41 | 0.012 | 0.529 |
| Fourth ventricle^3^ | 0.03 | 0.03 | 0.04 | 0.03 | 0.003 | 0.279 |
| Hypothalamus | 0.15 | 0.15 | 0.15 | 0.15 | 0.003 | 0.703 |
| Lateral ventricle^3^ | 0.61 | 0.59 | 0.61 | 0.58 | 0.018 | 0.457 |
| Left caudate | 0.39 | 0.39 | 0.40 | 0.38 | 0.011 | 0.147 |
| Left cortex | 28.20 | 28.04 | 29.18 | 28.13 | 0.644 | 0.248 |
| Left hippocampus | 0.49 | 0.48 | 0.49 | 0.48 | 0.010 | 0.965 |
| Left inferior colliculi^3^ | 0.12 | 0.12 | 0.12 | 0.12 | 0.003 | 0.412 |
| Left internal capsule^3^ | 0.90 | 0.89 | 0.91 | 0.88 | 0.023 | 0.389 |
| Left olfactory bulb^3^ | 1.96 | 1.93 | 1.96 | 1.99 | 0.050 | 0.514 |
| Left putamen-globus pallidus | 0.22 | 0.21 | 0.23 | 0.21 | 0.007 | 0.277 |
| Left superior colliculi^3^ | 0.28 | 0.28 | 0.28 | 0.28 | 0.006 | 0.555 |
| Medulla^3^ | 2.58 | 2.48 | 2.71 | 2.59 | 0.102 | 0.976 |
| Midbrain | 3.44 | 3.49 | 3.45 | 3.48 | 0.068 | 0.907 |
| Pons | 2.08 | 2.01 | 2.15 | 2.10 | 0.059 | 0.830 |
| Right caudate | 0.40 | 0.40 | 0.41 | 0.39 | 0.011 | 0.078 |
| Right cortex | 27.55 | 27.65 | 27.83 | 27.49 | 0.485 | 0.464 |
| Right hippocampus^3^ | 0.52 | 0.51 | 0.51 | 0.50 | 0.011 | 0.864 |
| Right inferior colliculi^3^ | 0.12 | 0.12 | 0.12 | 0.12 | 0.003 | 0.974 |
| Right internal capsule | 0.85 | 0.83 | 0.86 | 0.83 | 0.024 | 0.686 |
| Right olfactory bulb^3^ | 1.91 | 1.90 | 1.90 | 1.90 | 0.046 | 0.982 |
| Right putamen-globus pallidus | 0.20 | 0.19 | 0.20 | 0.19 | 0.006 | 0.967 |
| Right superior colliculi^3^ | 0.30 | 0.29 | 0.29 | 0.29 | 0.006 | 0.969 |
| Thalamus | 1.92 | 1.88 | 1.91 | 1.89 | 0.041 | 0.743 |
| Third ventricle^3^ | 0.04 | 0.03 | 0.04 | 0.04 | 0.002 | 0.399 |
| ^1^Data presented are least squares means and *P*-values from mixed model 2-way ANOVA.  ^2^Abbreviations: CON, control without supplementation; FL, 2’-FL supplementation; BI, Bi-26 administration; FLBI, 2’-FL supplementation and Bi-26 administration; SEM, standard error of mean.  ^3^Data transformation was necessary due to a violation of the homogeneity of variance assumption. | | | | | | |

| Supplemental Table 6. Axial diffusivity values (AD; x 10⁻³ /mm²/s) of pigs receiving milk replacer treatments differing in prebiotic supplementation^1^ | | | | |
| --- | --- | --- | --- | --- |
|  | **Main Effect Means** | |  | **Main Effect** |
| Region of Interest | **CON & BI** | **FL & FLBI** | **Pooled SEM^2^** | ***P*-value** |
| *Number of replicate pigs* | *25* | *21* | **-** | **-** |
| Cerebellum | 0.621 | 0.621 | 0.006 | 0.969 |
| Corpus callosum | 0.815 | 0.822 | 0.008 | 0.504 |
| Left caudate | 0.841 | 0.845 | 0.010 | 0.734 |
| Left hippocampus^3^ | 0.861 | 0.856 | 0.010 | 0.465 |
| Left internal capsule^3^ | 0.824 | 0.816 | 0.006 | 0.222 |
| Left side | 0.755 | 0.754 | 0.005 | 0.705 |
| Right caudate | 0.851 | 0.858 | 0.012 | 0.620 |
| Right hippocampus | 0.854 | 0.847 | 0.014 | 0.491 |
| Right internal capsule | 0.831 | 0.828 | 0.008 | 0.717 |
| Right side | 0.751 | 0.749 | 0.005 | 0.725 |
| Thalamus | 0.756 | 0.751 | 0.006 | 0.455 |
| White matter | 0.760 | 0.762 | 0.004 | 0.691 |
| Average AD mask | 0.729 | 0.730 | 0.007 | 0.876 |
| ^1^Data presented are least squares means and *P*-values from mixed model 2-way ANOVA.  ^2^Abbreviations: CON, control without supplementation; FL, 2’-FL supplementation; BI, Bi-26 administration; FLBI, 2’-FL supplementation and Bi-26 administration; SEM, standard error of mean.  ^3^Data transformation was necessary due to a violation of the homogeneity of variance assumption. | | | | |

| Supplemental Table 7. Axial diffusivity values (AD; x 10⁻³ /mm²/s) of pigs receiving milk replacer treatments differing in probiotic supplementation^1^ | | | | |
| --- | --- | --- | --- | --- |
|  | **Main Effect Means** | |  | **Main Effect** |
| Region of Interest | **CON & FL** | **BI & FLBI** | **Pooled SEM^2^** | ***P*-value** |
| *Number of replicate pigs* | *23* | *23* | **-** | **-** |
| Cerebellum | 0.618 | 0.624 | 0.006 | 0.349 |
| Corpus callosum | 0.813 | 0.824 | 0.008 | 0.263 |
| Left caudate | 0.844 | 0.842 | 0.010 | 0.882 |
| Left hippocampus^3^ | 0.857 | 0.859 | 0.010 | 0.841 |
| Left internal capsule^3^ | 0.813 | 0.827 | 0.006 | 0.025 |
| Left side | 0.752 | 0.757 | 0.005 | 0.238 |
| Right caudate | 0.851 | 0.858 | 0.012 | 0.602 |
| Right hippocampus | 0.846 | 0.855 | 0.014 | 0.409 |
| Right internal capsule | 0.828 | 0.831 | 0.008 | 0.600 |
| Right side | 0.746 | 0.753 | 0.005 | 0.139 |
| Thalamus | 0.752 | 0.755 | 0.006 | 0.704 |
| White matter | 0.757 | 0.766 | 0.004 | 0.047 |
| Average AD mask | 0.725 | 0.734 | 0.006 | 0.173 |
| ^1^Data presented are least squares means and *P*-values from mixed model 2-way ANOVA.  ^2^Abbreviations: CON, control without supplementation; FL, 2’-FL supplementation; BI, Bi-26 administration; FLBI, 2’-FL supplementation and Bi-26 administration; SEM, standard error of mean.  ^3^Data transformation was necessary due to a violation of the homogeneity of variance assumption. | | | | |

| Supplemental Table 8. Axial diffusivity values (AD; x 10⁻³ /mm²/s) of pigs assigned to factorial arrangement of prebiotic and probiotic supplementation^1^ | | | | | | |
| --- | --- | --- | --- | --- | --- | --- |
|  | **Interaction Means** | | | |  |  |
| Region of Interest | **CON** | **BI** | **FL** | **FLBI** | **Pooled SEM^2^** | **Interaction *P*-value** |
| *Number of replicate pigs* | *13* | *12* | *10* | *11* | *-* | *-* |
| Cerebellum | 0.616 | 0.626 | 0.619 | 0.622 | 0.008 | 0.584 |
| Corpus callosum | 0.801 | 0.830 | 0.825 | 0.818 | 0.011 | 0.073 |
| Left caudate | 0.839 | 0.842 | 0.849 | 0.841 | 0.014 | 0.683 |
| Left hippocampus^3^ | 0.851 | 0.871 | 0.864 | 0.847 | 0.012 | 0.025 |
| Left internal capsule^3^ | 0.811 | 0.837 | 0.814 | 0.817 | 0.008 | 0.082 |
| Left side | 0.748 | 0.762 | 0.755 | 0.752 | 0.006 | 0.063 |
| Right caudate | 0.844 | 0.859 | 0.858 | 0.857 | 0.016 | 0.550 |
| Right hippocampus | 0.843 | 0.865 | 0.849 | 0.844 | 0.016 | 0.198 |
| Right internal capsule | 0.822 | 0.840 | 0.834 | 0.823 | 0.009 | 0.042 |
| Right side | 0.742 | 0.759 | 0.749 | 0.748 | 0.007 | 0.096 |
| Thalamus | 0.750 | 0.761 | 0.754 | 0.748 | 0.008 | 0.165 |
| White matter | 0.751^a^ | 0.770^b^ | 0.762 | 0.762 | 0.006 | 0.039 |
| Average AD mask | 0.721 | 0.737 | 0.730 | 0.731 | 0.008 | 0.218 |
| ^ab^Superscript letters denote differences between the No Prebiotic: No Probiotic group and the No Prebiotic: Yes Probiotic group driven from the Dunnett’s test (*P* < 0.05).  ^1^Data presented are least squares means and *P*-values from mixed model 2-way ANOVA.  ^2^Abbreviations: CON, control without supplementation; FL, 2’-FL supplementation; BI, Bi-26 administration; FLBI, 2’-FL supplementation and Bi-26 administration; SEM, standard error of mean.  ^3^Data transformation was necessary due to a violation of the homogeneity of variance assumption. | | | | | | |

| Supplemental Table 9. Mean diffusivity values (MD; x 10⁻³ /mm²/s) of pigs receiving milk replacer treatments differing in prebiotic supplementation^1^ | | | | |
| --- | --- | --- | --- | --- |
|  | **Main Effect Means** | |  | **Main Effect** |
| Region of Interest | **CON & BI** | **FL & FLBI** | **Pooled SEM^2^** | ***P*-value** |
| *Number of replicate pigs* | *25* | *21* | **-** | **-** |
| Cerebellum | 0.494 | 0.495 | 0.005 | 0.882 |
| Corpus callosum | 0.611 | 0.620 | 0.006 | 0.182 |
| Left caudate | 0.618 | 0.618 | 0.006 | 0.942 |
| Left hippocampus | 0.632 | 0.625 | 0.007 | 0.354 |
| Left internal capsule | 0.490 | 0.487 | 0.003 | 0.490 |
| Left side | 0.536 | 0.536 | 0.004 | 0.893 |
| Right caudate^3^ | 0.652 | 0.657 | 0.008 | 0.508 |
| Right hippocampus | 0.622 | 0.616 | 0.009 | 0.463 |
| Right internal capsule | 0.493 | 0.490 | 0.004 | 0.617 |
| Right side | 0.533 | 0.535 | 0.003 | 0.667 |
| Thalamus^3^ | 0.572 | 0.570 | 0.005 | 0.656 |
| White matter | 0.539 | 0.539 | 0.004 | 0.971 |
| Average MD mask | 0.540 | 0.541 | 0.004 | 0.740 |
| ^1^Data presented are least squares means and *P*-values from mixed model 2-way ANOVA.  ^2^Abbreviations: CON, control without supplementation; FL, 2’-FL supplementation; BI, Bi-26 administration; FLBI, 2’-FL supplementation and Bi-26 administration; SEM, standard error of mean.  ^3^Data transformation was necessary due to a violation of the homogeneity of variance assumption. | | | | |

| Supplemental Table 10. Mean diffusivity values (MD; x 10⁻³ /mm²/s) of pigs receiving milk replacer treatments differing in probiotic supplementation^1^ | | | | |
| --- | --- | --- | --- | --- |
|  | **Main Effect Means** | |  | **Main Effect** |
| Region of Interest | **CON & FL** | **BI & FLBI** | **Pooled SEM^2^** | ***P*-value** |
| *Number of replicate pigs* | *23* | *23* | **-** | **-** |
| Cerebellum | 0.493 | 0.497 | 0.006 | 0.438 |
| Corpus callosum | 0.612 | 0.619 | 0.006 | 0.282 |
| Left caudate | 0.617 | 0.619 | 0.006 | 0.806 |
| Left hippocampus | 0.628 | 0.628 | 0.007 | 0.971 |
| Left internal capsule | 0.485 | 0.492 | 0.003 | 0.140 |
| Left side | 0.536 | 0.537 | 0.004 | 0.675 |
| Right caudate^3^ | 0.657 | 0.652 | 0.008 | 0.591 |
| Right hippocampus | 0.615 | 0.622 | 0.009 | 0.341 |
| Right internal capsule | 0.488 | 0.495 | 0.004 | 0.238 |
| Right side | 0.532 | 0.535 | 0.003 | 0.363 |
| Thalamus^3^ | 0.571 | 0.571 | 0.005 | 0.898 |
| White matter | 0.536 | 0.541 | 0.004 | 0.237 |
| Average MD mask | 0.538 | 0.544 | 0.004 | 0.186 |
| ^1^Data presented are least squares means and *P*-values from mixed model 2-way ANOVA.  ^2^Abbreviations: CON, control without supplementation; FL, 2’-FL supplementation; BI, Bi-26 administration; FLBI, 2’-FL supplementation and Bi-26 administration; SEM, standard error of mean.  ^3^Data transformation was necessary due to a violation of the homogeneity of variance assumption. | | | | |

| Supplemental Table 11. Mean diffusivity values (MD; x 10⁻³ /mm²/s) of pigs assigned to factorial arrangement of prebiotic and probiotic supplementation^1^ | | | | | | |
| --- | --- | --- | --- | --- | --- | --- |
|  | **Interaction Means** | | | |  |  |
| Region of Interest | **CON** | **BI** | **FL** | **FLBI** | **Pooled SEM^2^** | **Interaction *P*-value** |
| *Number of replicate pigs* | *13* | *12* | *10* | *11* | *-* | *-* |
| Cerebellum | 0.491 | 0.498 | 0.494 | 0.496 | 0.007 | 0.714 |
| Corpus callosum | 0.609 | 0.613 | 0.615 | 0.624 | 0.007 | 0.730 |
| Left caudate | 0.616 | 0.620 | 0.618 | 0.618 | 0.008 | 0.816 |
| Left hippocampus | 0.626 | 0.637 | 0.631 | 0.619 | 0.009 | 0.098 |
| Left internal capsule | 0.480^a^ | 0.500^b^ | 0.490 | 0.484 | 0.005 | 0.006 |
| Left side | 0.532 | 0.541 | 0.539 | 0.533 | 0.004 | 0.018 |
| Right caudate^3^ | 0.652 | 0.652 | 0.663 | 0.651 | 0.010 | 0.433 |
| Right hippocampus | 0.616 | 0.627 | 0.614 | 0.618 | 0.011 | 0.679 |
| Right internal capsule | 0.485 | 0.501 | 0.492 | 0.489 | 0.006 | 0.112 |
| Right side | 0.529 | 0.537 | 0.536 | 0.534 | 0.004 | 0.147 |
| Thalamus^3^ | 0.568 | 0.576 | 0.573 | 0.567 | 0.006 | 0.140 |
| White matter | 0.533 | 0.544 | 0.540 | 0.537 | 0.005 | 0.069 |
| Average MD mask | 0.534 | 0.546 | 0.541 | 0.542 | 0.006 | 0.227 |
| ^ab^Superscript letters denote differences between the No Prebiotic: No Probiotic group and the No Prebiotic: Yes Probiotic group driven from the Dunnett’s test (*P* < 0.05).  ^1^Data presented are least squares means and *P*-values from mixed model 2-way ANOVA.  ^2^Abbreviations: CON, control without supplementation; FL, 2’-FL supplementation; BI, Bi-26 administration; FLBI, 2’-FL supplementation and Bi-26 administration; SEM, standard error of mean.  ^3^Data transformation was necessary due to a violation of the homogeneity of variance assumption. | | | | | | |

| Supplemental Table 12. Radial diffusivity values (RD; x 10⁻³ /mm²/s) of pigs receiving milk replacer treatments differing in prebiotic supplementation^1^ | | | | |
| --- | --- | --- | --- | --- |
|  | **Main Effect Means** | |  | **Main Effect** |
| Region of Interest | **CON & BI** | **FL & FLBI** | **Pooled SEM^2^** | ***P*-value** |
| *Number of replicate pigs* | *25* | *21* | **-** | **-** |
| Cerebellum | 0.431 | 0.433 | 0.005 | 0.789 |
| Corpus callosum^3^ | 0.508 | 0.516 | 0.007 | 0.366 |
| Left caudate | 0.503 | 0.504 | 0.005 | 0.971 |
| Left hippocampus^3^ | 0.513 | 0.511 | 0.006 | 0.780 |
| Left internal capsule^3^ | 0.329 | 0.327 | 0.005 | 0.961 |
| Left side | 0.427 | 0.428 | 0.003 | 0.862 |
| Right caudate | 0.553 | 0.557 | 0.010 | 0.505 |
| Right hippocampus | 0.506 | 0.501 | 0.007 | 0.487 |
| Right internal capsule^3^ | 0.329 | 0.328 | 0.005 | 0.819 |
| Right side | 0.425 | 0.427 | 0.003 | 0.516 |
| Thalamus | 0.479 | 0.480 | 0.004 | 0.944 |
| White matter | 0.424 | 0.427 | 0.003 | 0.308 |
| Average RD mask | 0.445 | 0.445 | 0.004 | 0.974 |
| ^1^Data presented are least squares means and *P*-values from mixed model 2-way ANOVA.  ^2^Abbreviations: CON, control without supplementation; FL, 2’-FL supplementation; BI, Bi-26 administration; FLBI, 2’-FL supplementation and Bi-26 administration; SEM, standard error of mean.  ^3^Data transformation was necessary due to a violation of the homogeneity of variance assumption. | | | | |

| Supplemental Table 13. Radial diffusivity values (RD; x 10⁻³ /mm²/s) of pigs receiving milk replacer treatments differing in probiotic supplementation^1^ | | | | |
| --- | --- | --- | --- | --- |
|  | **Main Effect Means** | |  | **Main Effect** |
| Region of Interest | **CON & FL** | **BI & FLBI** | **Pooled SEM^2^** | ***P*-value** |
| *Number of replicate pigs* | *23* | *23* | **-** | **-** |
| Cerebellum | 0.430 | 0.433 | 0.005 | 0.528 |
| Corpus callosum^3^ | 0.508 | 0.516 | 0.007 | 0.249 |
| Left caudate | 0.504 | 0.504 | 0.005 | 0.991 |
| Left hippocampus^3^ | 0.511 | 0.513 | 0.006 | 0.930 |
| Left internal capsule^3^ | 0.327 | 0.330 | 0.005 | 0.557 |
| Left side | 0.427 | 0.428 | 0.003 | 0.867 |
| Right caudate | 0.561 | 0.549 | 0.010 | 0.103 |
| Right hippocampus | 0.500 | 0.506 | 0.007 | 0.386 |
| Right internal capsule^3^ | 0.327 | 0.331 | 0.005 | 0.372 |
| Right side | 0.425 | 0.427 | 0.003 | 0.533 |
| Thalamus | 0.479 | 0.480 | 0.004 | 0.951 |
| White matter | 0.425 | 0.427 | 0.003 | 0.569 |
| Average RD mask | 0.442 | 0.448 | 0.004 | 0.126 |
| ^1^Data presented are least squares means and *P*-values from mixed model 2-way ANOVA.  ^2^Abbreviations: CON, control without supplementation; FL, 2’-FL supplementation; BI, Bi-26 administration; FLBI, 2’-FL supplementation and Bi-26 administration; SEM, standard error of mean.  ^3^Data transformation was necessary due to a violation of the homogeneity of variance assumption. | | | | |

| Supplemental Table 14. Radial diffusivity values (RD; x 10⁻³ /mm²/s) of pigs assigned to factorial arrangement of prebiotic and probiotic supplementation^1^ | | | | | | |
| --- | --- | --- | --- | --- | --- | --- |
|  | **Interaction Means** | | | |  |  |
| Region of Interest | **CON** | **BI** | **FL** | **FLBI** | **Pooled SEM^2^** | **Interaction *P*-value** |
| *Number of replicate pigs* | *13* | *12* | *10* | *11* | *-* | *-* |
| Cerebellum | 0.429 | 0.433 | 0.432 | 0.434 | 0.007 | 0.801 |
| Corpus callosum^3^ | 0.508 | 0.508 | 0.508 | 0.524 | 0.010 | 0.489 |
| Left caudate | 0.505 | 0.502 | 0.502 | 0.505 | 0.008 | 0.633 |
| Left hippocampus^3^ | 0.507 | 0.519 | 0.515 | 0.506 | 0.008 | 0.237 |
| Left internal capsule^3^ | 0.323 | 0.336 | 0.330 | 0.324 | 0.007 | 0.150 |
| Left side | 0.424 | 0.431 | 0.431 | 0.425 | 0.004 | 0.013 |
| Right caudate | 0.556 | 0.550 | 0.566 | 0.549 | 0.011 | 0.437 |
| Right hippocampus | 0.503 | 0.508 | 0.497 | 0.505 | 0.009 | 0.790 |
| Right internal capsule^3^ | 0.323 | 0.336 | 0.330 | 0.326 | 0.007 | 0.216 |
| Right side | 0.422 | 0.428 | 0.429 | 0.426 | 0.004 | 0.167 |
| Thalamus | 0.476 | 0.482 | 0.483 | 0.477 | 0.006 | 0.271 |
| White matter | 0.422 | 0.427 | 0.428 | 0.426 | 0.003 | 0.135 |
| Average RD mask | 0.441 | 0.450 | 0.443 | 0.447 | 0.005 | 0.499 |
| ^1^Data presented are least squares means and *P*-values from mixed model 2-way ANOVA.  ^2^Abbreviations: CON, control without supplementation; FL, 2’-FL supplementation; BI, Bi-26 administration; FLBI, 2’-FL supplementation and Bi-26 administration; SEM, standard error of mean.  ^3^Data transformation was necessary due to a violation of the homogeneity of variance assumption. | | | | | | |

| Supplemental Table 15. Fractional anisotropy values (FA; arbitrary units) of pigs receiving milk replacer treatments differing in prebiotic supplementation^1^ | | | | |
| --- | --- | --- | --- | --- |
|  | **Main Effect Means** | |  | **Main Effect** |
| Region of Interest | **CON & BI** | **FL & FLBI** | **Pooled SEM^2^** | ***P*-value** |
| *Number of replicate pigs* | *25* | *21* | **-** | **-** |
| Cerebellum | 0.237 | 0.237 | 0.003 | 0.782 |
| Corpus callosum^3^ | 0.327 | 0.325 | 0.006 | 0.810 |
| Left caudate | 0.320 | 0.325 | 0.007 | 0.616 |
| Left hippocampus | 0.331 | 0.325 | 0.006 | 0.359 |
| Left internal capsule | 0.562 | 0.562 | 0.006 | 0.970 |
| Left side | 0.360 | 0.359 | 0.002 | 0.564 |
| Right caudate^3^ | 0.288 | 0.288 | 0.009 | 0.923 |
| Right hippocampus | 0.329 | 0.332 | 0.005 | 0.628 |
| Right internal capsule | 0.542 | 0.535 | 0.006 | 0.406 |
| Right side | 0.359 | 0.358 | 0.002 | 0.795 |
| Thalamus | 0.301 | 0.300 | 0.003 | 0.826 |
| White matter | 0.370 | 0.369 | 0.002 | 0.654 |
| Average FA mask | 0.325 | 0.323 | 0.001 | 0.145 |
| ^1^Data presented are least squares means and *P*-values from mixed model 2-way ANOVA.  ^2^Abbreviations: CON, control without supplementation; FL, 2’-FL supplementation; BI, Bi-26 administration; FLBI, 2’-FL supplementation and Bi-26 administration; SEM, standard error of mean.  ^3^Data transformation was necessary due to a violation of the homogeneity of variance assumption. | | | | |

| Supplemental Table 16. Fractional anisotropy (FA; arbitrary units) of pigs receiving milk replacer treatments differing in probiotic supplementation^1^ | | | | |
| --- | --- | --- | --- | --- |
|  | **Main Effect Means** | |  | **Main Effect** |
| Region of Interest | **CON & FL** | **BI & FLBI** | **Pooled SEM^2^** | ***P*-value** |
| *Number of replicate pigs* | *23* | *23* | **-** | **-** |
| Cerebellum | 0.237 | 0.237 | 0.003 | 0.891 |
| Corpus callosum^3^ | 0.325 | 0.327 | 0.006 | 0.733 |
| Left caudate | 0.326 | 0.320 | 0.007 | 0.441 |
| Left hippocampus | 0.331 | 0.325 | 0.005 | 0.335 |
| Left internal capsule | 0.563 | 0.561 | 0.006 | 0.789 |
| Left side | 0.361 | 0.359 | 0.002 | 0.373 |
| Right caudate^3^ | 0.282 | 0.294 | 0.009 | 0.106 |
| Right hippocampus | 0.332 | 0.330 | 0.005 | 0.743 |
| Right internal capsule | 0.537 | 0.540 | 0.006 | 0.637 |
| Right side | 0.359 | 0.358 | 0.002 | 0.964 |
| Thalamus | 0.302 | 0.300 | 0.003 | 0.493 |
| White matter | 0.370 | 0.369 | 0.002 | 0.857 |
| Average FA mask | 0.325 | 0.324 | 0.001 | 0.670 |
| ^1^Data presented are least squares means and *P*-values from mixed model 2-way ANOVA.  ^2^Abbreviations: CON, control without supplementation; FL, 2’-FL supplementation; BI, Bi-26 administration; FLBI, 2’-FL supplementation and Bi-26 administration; SEM, standard error of mean.  ^3^Data transformation was necessary due to a violation of the homogeneity of variance assumption. | | | | |

| Supplemental Table 17. Fractional anisotropy values (FA; arbitrary units) of pigs assigned to factorial arrangement of prebiotic and probiotic supplementation^1^ | | | | | | |
| --- | --- | --- | --- | --- | --- | --- |
|  | **Interaction Means** | | | |  |  |
| Region of Interest | **CON** | **BI** | **FL** | **FLBI** | **Pooled SEM^2^** | **Interaction *P*-value** |
| *Number of replicate pigs* | *13* | *12* | *10* | *11* | *-* | *-* |
| Cerebellum | 0.237 | 0.237 | 0.236 | 0.237 | 0.003 | 0.928 |
| Corpus callosum^3^ | 0.316 | 0.338 | 0.333 | 0.316 | 0.009 | 0.030 |
| Left caudate | 0.323 | 0.317 | 0.330 | 0.321 | 0.010 | 0.872 |
| Left hippocampus | 0.334 | 0.328 | 0.328 | 0.322 | 0.007 | 0.965 |
| Left internal capsule | 0.565 | 0.559 | 0.560 | 0.563 | 0.009 | 0.562 |
| Left side | 0.363 | 0.358 | 0.359 | 0.360 | 0.003 | 0.152 |
| Right caudate^3^ | 0.282 | 0.294 | 0.282 | 0.294 | 0.011 | 0.955 |
| Right hippocampus | 0.325 | 0.333 | 0.339 | 0.326 | 0.007 | 0.112 |
| Right internal capsule | 0.541 | 0.543 | 0.533 | 0.538 | 0.008 | 0.885 |
| Right side | 0.360 | 0.358 | 0.357 | 0.359 | 0.003 | 0.427 |
| Thalamus | 0.304 | 0.298 | 0.300 | 0.301 | 0.004 | 0.279 |
| White matter | 0.371 | 0.369 | 0.368 | 0.370 | 0.003 | 0.303 |
| Average FA mask | 0.325 | 0.326 | 0.324 | 0.323 | 0.002 | 0.416 |
| ^1^Data presented are least squares means and *P*-values from mixed model 2-way ANOVA.  ^2^Abbreviations: CON, control without supplementation; FL, 2’-FL supplementation; BI, Bi-26 administration; FLBI, 2’-FL supplementation and Bi-26 administration; SEM, standard error of mean.  ^3^Data transformation was necessary due to a violation of the homogeneity of variance assumption. | | | | | | |
